# Human-Avatar Symbiosis for the Treatment of Auditory Verbal Hallucinations in Schizophrenia through Virtual/Augmented Reality and Brain-Computer Interfaces

**DOI:** 10.3389/fninf.2017.00064

**Published:** 2017-11-21

**Authors:** Antonio Fernández-Caballero, Elena Navarro, Patricia Fernández-Sotos, Pascual González, Jorge J. Ricarte, José M. Latorre, Roberto Rodriguez-Jimenez

**Affiliations:** ^1^Departamento de Sistemas Informáticos, Universidad de Castilla-La Mancha, Albacete, Spain; ^2^Centro de Investigación Biomédica en Red de Salud Mental (CIBERSAM), Madrid, Spain; ^3^Instituto de Investigación Hospital 12 de Octubre (imas12), Madrid, Spain; ^4^Departamento de Psicología, Universidad de Castilla-La Mancha, Albacete, Spain

**Keywords:** human-avatar symbiosis, virtual reality, augmented reality, brain-computer interfaces, auditory verbal hallucinations

## Abstract

This perspective paper faces the future of alternative treatments that take advantage of a social and cognitive approach with regards to pharmacological therapy of auditory verbal hallucinations (AVH) in patients with schizophrenia. AVH are the perception of voices in the absence of auditory stimulation and represents a severe mental health symptom. Virtual/augmented reality (VR/AR) and brain computer interfaces (BCI) are technologies that are growing more and more in different medical and psychological applications. Our position is that their combined use in computer-based therapies offers still unforeseen possibilities for the treatment of physical and mental disabilities. This is why, the paper expects that researchers and clinicians undergo a pathway toward human-avatar symbiosis for AVH by taking full advantage of new technologies. This outlook supposes to address challenging issues in the understanding of non-pharmacological treatment of schizophrenia-related disorders and the exploitation of VR/AR and BCI to achieve a real human-avatar symbiosis.

## 1. Introduction

Nowadays, mental, neurological, and substance-use (MNS) disorders are one main problem in developed countries. Schizophrenia, epilepsy, dementia, and other MNS disorders constitute 13% of the global burden of disease, surpassing both cardiovascular disease and cancer (Collins et al., 2011). Schizophrenia belongs to the group of grand challenges in global mental health. This mental disorder is characterized by abnormal social behavior and failure to recognize what is real. It has different symptoms such as unclear or confused thinking, reduced social engagement and emotional expression, false beliefs (delusions), and hallucinations, especially auditory verbal hallucinations (AVH).

Among all symptoms, AVH are one of the most frequent as between 50 and 80% of people with schizophrenia suffer it. AVH are perception of voices in the absence of auditory stimulation. Hallucinations, along with delusional ideas, are the most characteristic symptoms of this disorder. The ensuing distress is often high and results in severe work, family social dysfunction and suicide attempts (Cheung et al., 2007; Wong et al., 2007). Antipsychotic medication is effective in most cases but is often accompanied by side effects such as weight gain, somnolence, hyperprolactinaemia, and dystonia and other movement disorders. As a result, patients may refuse antipsychotic medication or withdraw treatment (Byerly et al., 2007). In addition, 25–30% of patients experience AVH that are unresponsive to antipsychotic medication (Shergill et al., 1998; Harrow et al., 2014).

In this context, given the relative stagnation in the last decades of pharmacological alternatives focused on the dopaminergic system, proposals based on non-pharmacological strategies have acquired new value (Wykes et al., 2008). On the one hand, cognitive-behavioral approaches centred on cognitive rehabilitation (both neurocognition and social cognition) are exhibiting significant growth, with many articles and meta-analyses showing their efficacy (McGurk et al., 2007). Another type of non-pharmacological approaches is based on cognitive-behavioral psychotherapy in so-called high-risk subjects, trying to prevent the development of psychotic disorder. The results obtained to date are promising (McGorry et al., 2013), especially considering that we would be talking about a level that is still preventive. Finally, other approaches refer to the non-pharmacological treatment of productive psychotic symptoms (mainly hallucinations and delusions) that are resistant to pharmacological treatment or that respond only partially. Some cognitive behavioral therapies (CBT) have been developed to treat these refractory symptoms (Chadwick et al., 1996; Sensky et al., 2000; Kråkvik et al., 2013; Birchwood et al., 2014). The published works on these approaches show efficacy, as well as good acceptance by the patients (van der Gaag et al., 2014).

These facts highlight the need of providing both specialists and patients with alternative treatments that take advantage of a cognitive and social approach. Therefore, any perspective for the development of new treatments for AVH should face two issues: creation of social environments similar to the real world where the patient is guided toward successful social situations, and, real-time monitoring of cerebral areas activated by the patient while living these situations to obtain information about the neurological effects of his/her decisions.

## 2. Toward human-avatar symbiosis for auditory verbal hallucinations

Nowadays, the treatment of patients with schizophrenia suffering AVH consists of face-to-face sessions and controlled use of antipsychotic drugs. As Harrow et al. (2014) have shown that an important number of these patients do not respond to the treatment, novel alternatives are demanded by patients, families, clinicians and society. Our perspective is to exploit computer-mediated therapies, more concretely, therapies that are carried out in virtual environments so that the interaction with the patient is totally under control and the patient is empowered to participate in the management of his/her disorder through the use of brain-computer interfaces (BCI). These computer-mediated therapies are based in CBT.

First, our outlook is based on previous studies that promote the use of virtual reality (VR) to create controlled social environments to carry out the treatment. One such approach has developed a novel therapy based on a computer program, which enables patients to create avatars that match the entities, human or non-human, that they believe are interacting with them through voices (Leff et al., 2014). This therapy is designed to encourage the dialogue between patient and avatar so that he/she could control it. To facilitate this dialogue, a voice-morphing program and virtual reality environment are used, so that the patients constructs an appropriate avatar. Finally, therapists use the constructed avatars to speak with their patients. The results of this study show that many patients were able to stand up to their AVH but not all patients may be able to face their persecutor during therapy (Leff et al., 2014). Moreover, the very distressing aspect of AVH is that they usually occur in patients' daily-life situations. This has led to consider the need of developing an avatar of the virtual therapist (instead of the emitter of voices) that assists a patient whenever hallucinations appear. This implies to develop and reach a good relationship between the patient and his/her personal avatar and to be certain that the avatar's interventions are experienced as positive and helpful. For that purpose, the use of subjective measures from the patients combined with more objective and neurophysiological measures is important and these challenges require the use of advanced artificial intelligence (AI) technologies.

**Figure 1:**
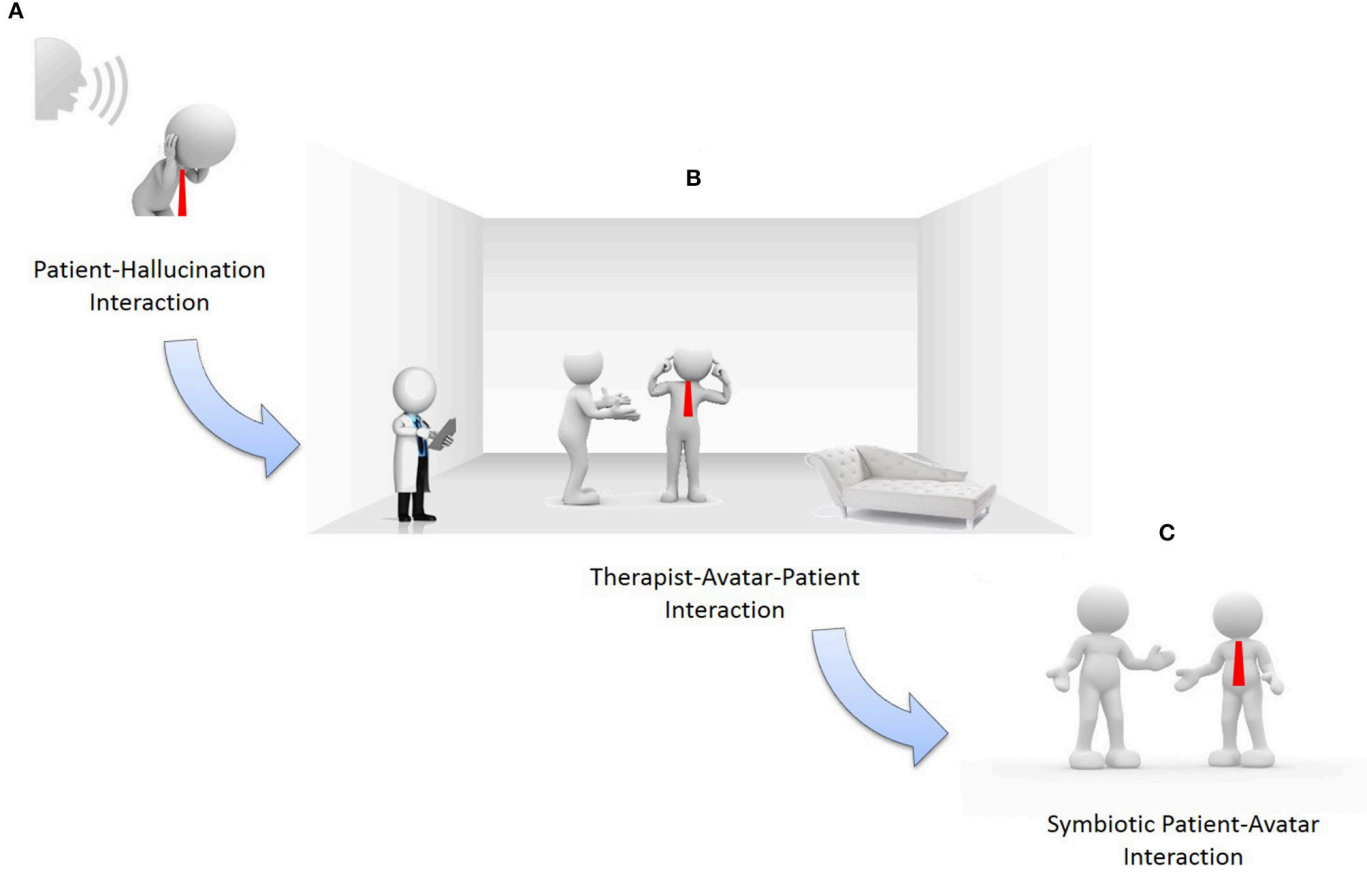
Evolving to symbiotic human-avatar interaction. **(A)** patient-hallucinations interaction; **(B)** therapist-avatar-patient interaction; **(C)** symbiotic patient-avatar interaction.

Second, to take full advantage of computer-based therapies, a symbiotic human-avatar interaction should be achieved. Symbiosis is defined as “a close and prolonged relation between two or more organisms of different species that benefits each of the members.” Until now, human-machine symbiosis (HMS) is being applied timidly in medical sciences to improve quality of life (Jolliffe, 2012). Several technologies as electrophysiology, especially BCI exploiting electroencephalography (EEG), achieve such HMS. BCI are used in our viewpoint as objective neurophysiological measures of patient-therapist and patient-avatar interaction. The development of this symbiotic human-avatar interaction is performed by analysing, designing and evaluating the problem throughout three different stages (see Figure 1).

Interaction “subject-psychotherapist.” As shown in Figure 1A, the subject is suffering auditory hallucinations. Firstly, automatic recordings of psychotherapist-patient sessions are carried out. These valuable conversations describe patients' auditory hallucinations, necessary for designing avatars as well as virtual and augmented reality (VR/AR) environments. Moreover, during this first phase the patient is monitored to record his/her neurophysiological measures in order to better understand his/her behavior.

Interaction “subject-psychotherapist-avatar.” During this second stage, once VR/AR environments and customized avatars have been developed according to the patient' auditory hallucinations, the process that enables psychotherapists to control the avatar-patient conversations following the pre-defined scripts identified is ready (see Figure 1B). This stage is critical for future patient-avatar symbiosis. The psychotherapist controls the avatars by using the most suitable scripts per the patient' reactions in the VR/AR environments. Simultaneously, patient and therapist are monitored using BCI to record their neurophysiological measures to provide feedback to the learning/adaptation process in charge of controlling the avatars' behavior. This facilitates that the avatar's “brain” conforms, step by step, to the needs of the patient's social-affective relations. Simultaneously, the psychotherapist's know-how (knowledge and professional experience) is recorded. This continuous learning/adaptation process incrementally increases the symbiotic human-avatar interaction during different sessions.

Symbiotic interaction “subject-avatar.” A real subject-avatar symbiosis in VR/AR environments is the final goal of our standpoint (see Figure 1C). This interaction is symbiotic because both avatar and subject evolve together as the avatar can both learn about and interact with the subject. This learning ability bases on a beliefs-desires-intentions (BDI) model facilitating the avatar's adaptability per the subject's needs, environment, interaction, etc. Beliefs describe information about the patient's world, that is, the world of auditory hallucinations. The avatar assumes beliefs of both patient and psychotherapist. These beliefs are analyzed by the avatar considering the cerebral activity of both patient and psychotherapist. Desires, also known as goals, are the tasks assigned to the avatar. The avatar assumes the psychotherapist's desires while learning the patient's reactions and new goals, everything perceived from neuronal stimuli, to drive the patient to a positive socio-affective state. Intentions are action sequences that the avatar should carry out. Every intention is implemented as a stack of plans. Intentions consider the scripts that the psychotherapist designed during the psychotherapy sessions. These scripts are automatically modified during the neuronal feedback process.

As has been stated, one main cornerstone of the proposal is VR/AR exploitation. This technology controls the social environment where patient-therapist interaction is realized to provide the patient with necessary safe environments. For this reason, the system should provide a VR/AR editor and runtime for:

1. Designing avatars that represent the characters described in hallucinations.
2. Specifying scripts for psychotherapists to guide their conversations with patients by means of avatars.
3. Running VR/AR environments where avatar-patient experiments are accomplished.

BCI are another key element. This technology facilitates to characterize neural mechanisms and social interaction of patients and specialists in real-time while they interact in VR/AR environments. This enables patients to achieve cerebral self-regulation through neurological feedback. Moreover, exploitation of BCI emerges as a more natural approach than alternatives such as neuroimaging because they are much less invasive and more cost-effective.

## 3. Most important challenges in human-avatar symbiosis for auditory verbal hallucinations

### 3.1. In schizophrenia

The usual treatment of patients with schizophrenia involves using antipsychotics in combination with psychotherapy and rehabilitation programs and aims to help patients reduce illness' symptoms, improve social competence and quality of life. However, despite appropriate treatments some patients remain severely impaired by drug-resistant symptoms such as resistant auditory hallucinations. Specific CBT programs (Chadwick et al., 1996; Sensky et al., 2000; Kråkvik et al., 2013; Birchwood et al., 2014) have been developed with a focus on resistant hallucinations and have enabled patients to gain more control over them (van der Gaag et al., 2014).

The recent development of avatar of voices for treatment of auditory hallucinations represent a remarkable step in psychotherapy progress and is considered a revolutionary therapeutic innovation in the field of psychiatry (Craig et al., 2015). The use of Artificial Intelligence (AI) in the field psychotherapy for psychosis is new, emerging and at the same time highly challenging so that crucial improvement is needed to design avatars that best suit to patients. For that purpose, a broader expertise from AI is needed to guide the development and to improve quality of those avatars.

Our perspective proposes to add three critical and substantial improvements to existing avatars. First, the possibility for the avatar to learn from interactions between therapist and patient and between avatar and patient, which opens new options not envisioned by therapist or patient. Second, the use of new techniques in the VR field such as holograms, mirrors and even fog to visualize avatars and environments, providing a more controllable environment, as well as exploitation of haptic devices to improve sense of reality (Basdogan et al., 2000). Third, the use of BCI adds information to check the patient' acceptability and tolerance of the avatar. In fact, the lack of insight into illness and the difficulty for patients to express their own emotions and interpret those of others is a critical characteristic of schizophrenia and the solution of BCI measures during therapy provides objective data to complement subjective patients' reports. For these reasons our picture includes major steps in developing this challenging and emerging domain of schizophrenia treatment.

### 3.2. In virtual/augmented reality

VR and AR are found highly complementary for psychotherapies. Usually, therapeutic intervention is carried out in controlled social environments (therapist and subject) so that the transfer of therapies to real-life social situations are quite difficult. However, VR simulates real experiences that even provoke physiological reactions. This is thanks to its capabilities of immersion (user' self-awareness is reduced as s/he interacts) and presence (perception of virtual as real environment). An additional advantage of using virtual environments is that subjects have the virtual situation under control, reducing and even preventing anxiety. Moreover, AR facilitates that virtual objects overlap the real world as they were part of it.

Despite there are some studies about the suitability of using VR/AR to deal some aspects of schizophrenia such as improvement of social abilities for role-playing (Pogorelc et al., 2011), improvement of social cognition (Peyroux and Franck, 2014) and motivation for re-employment (Tsang and Man, 2013), there are no VR/AR tools for a holistic treatment of schizophrenia. Nevertheless, VR peripherals allow visualization of unlimited virtual environments. Firstly, they enable to simulate light and sound conditions of a room without physically building it or buying devices that produce such conditions. Secondly, they enable to simulate not only one room, but several interconnected spaces extending the experimentation possibilities. For this reason, the construction of immersive virtual environments is envisioned, which would offer a surrounding experience, both visual and auditory, and minimally intrusive and maximally transparent to the user. This objective implies several challenges.

One is simulating virtual environments that match color and intensity maps of real light bulbs and audio systems. Moreover, new stimuli, as those associated with haptic sense, such as vibrotactile stimuli (Martínez et al., 2014), should be explored to increase the environments' sensation of reality. Another challenge is that the user should wear as less devices as possible, which in practice means to consider more complex devices than conventional head-mounted displays. It leads to the design of projection screens such as a CAVE-like set up (Cruz-Neira et al., 1992). The use of holograms (Maimone et al., 2017), mirrors (Jang et al., 2014) and even fog (Martinez Plasencia et al., 2014) to visualize virtual images opens new ways of interaction and immersion, enabling to combine the representation of virtual and real objects and also the patient' resemblance. Finally, integration of BCI devices to handle a virtual avatar opens new ways of interaction in these environments.

### 3.3. In brain-computer interfaces

Affective computing (AC) is defined as “computing that relates to, arises from, or deliberately influences emotions” (Picard, 1997). AC focuses on sensing human affective states, modeling processes involved in affect, synthesizing emotional expressions and behaviors, and interacting between human and machine according to the user' affective state (Mühl et al., 2014). Affective BCI is a subfield of AC that attempts to develop methods and to design devices capable of detecting affective states from neurophysiological signals, and use this information to improve user interaction as an implicit affective tagging method (Koelstra et al., 2012). Research in this domain is highly interdisciplinary, using theories and methods from psychology, neuroscience, AI and human-machine interfaces (HMI) to induce, measure, and detect affective states. Neurophysiological signals are closer to the origin of affective states than physiological signals such as heart rate, skin conductance and muscle tension, although this advantage is mitigated by difficulties in recording brain signals in real-world settings and interpreting them in a participant-independent manner. On the other hand, neural signals are just another modality that supplements the visual or auditory channel. However, it is less dependent on overt behavior, less susceptible to deception, although requiring more intrusive sensors. Recent works have included affective BCI systems for entertainment, life-style and ergonomic applications (Zander and Kothe, 2011).

The typical BCI approach is stimulus-independent passive BCI, which includes general affect sensing for applications in HMI scenarios where adapting an application per a user' state is important. Information that identifies a user' affective states is used to adapt the behavior of an application to keep the user satisfied or engaged. For example, some studies found neurophysiological responses in theta and alpha frequency bands to differentiate between episodes of frustrating and normal game play (Shergill et al., 2013) or even musical emotion regulation (Fernández-Sotos et al., 2016; Martínez-Rodrigo et al., 2017).

In this panorama, state-of-the-art in affective BCIs advances in multiple directions. First, reliable EEG-based correlates of affective and cognitive states related to creative industrial applications are a big challenge. Once these reliable indicators are determined, the effect of contextual factors should be studied. This is especially relevant for robust affect detecting methods in the context of multimodal interaction and stimulation. For use in a company setting these methods need to be transferred from lab to real-life context of use. And finally, methods must be extended to a hybrid affect detection approach using other physiological sensors and data, requiring advanced fusion techniques (Castillo et al., 2016; Fernández-Caballero et al., 2016).

## 4. Discussion

During the last decades, many efforts have been developed to help patients with schizophrenia improve their symptoms, and more recently their functionality in real life and quality of life. Pharmacological approaches focused on the dopaminergic system have demonstrated a clear efficacy, but this is not the case for all patients. In this context, the development of non-pharmacological strategies has gained special relevance. Specifically, CBT for the management of refractory auditory hallucinations has gained a renewed interest in applying new technologies (creation of avatars) to previously developed psychotherapeutic models of demonstrated efficacy.

However, this domain is at its early beginning and there are only a limited number of research teams working in the development of first approximations to this therapy. Due to first promising results, more inputs are needed to encourage creativeness in this emerging domain and to strengthen scientific competitiveness and further support of companies interested in this domain. Also, therapists and patients all over the world are demanding new approaches to face this highly incidence illness and companies would be happy to develop and put into the global market the developed technical solutions.

“Human-Avatar Symbiosis for AVH through Integration of VR/AR and Brain-Computer Interfaces” offers important expected impacts. These could be: efficient cognitive therapies for symptoms of schizophrenia based on VR/AR and BCI, increase in quality of life, well-being and social integration of people with schizophrenia, software able to communicate brain and virtual atmospheres, and reduction in social and economic costs of mental illness. From a clinical point of view, we cannot forget the possibility of secondary effects in the patients of any therapeutic intervention (not only in pharmacological ones). Preliminary results are very positive in this regard, and the acceptability of this kind of therapy by patients is high. Nevertheless, it will be necessary to conduct clinical studies to probe acceptability of patients and occurrence of secondary effects.

Our adaptive approach leads to a first vision where an avatar-based therapy for schizophrenia can be tailored rapidly by the therapist. This provides a new level of innovation in therapies as a computer system offers adaptation similar to a personal therapy session with a patient. Our viewpoint is that technological inflexibility should not impede therapy quality. This also leads to new knowledge on how such flexible systems can aid in AVH therapy.

Moreover, such pack of solutions would create great impact among citizens. In relation to life quality, our approach generates a new non-invasive and drug independent therapy for patients with schizophrenia, with special indication to those with AVH. The therapy eventually mitigates these symptoms and finally AVH could disappear. The proposed treatment with avatars could even reduce the use of antipsychotic drugs doses and their secondary effects, as well as the global cost including economic and social impacts. In addition, as most patients suffering AVH avoid social interaction due to illness, even with family, friends or colleagues, our perspective contributes to encourage the patients' sociability, which results in functionality and life quality improvements.

## Author contributions

All authors listed have made a substantial, direct and intellectual contribution to the work, and approved it for publication.

### Conflict of interest statement

RR-J has been a consultant for, spoken in activities of, or received grants from Instituto de Salud Carlos III, Fondo de Investigación Sanitaria (FIS), Centro de Investigación Biomédica en Red de Salud Mental (CIBERSAM), Madrid Regional Government (S2010/ BMD-2422 AGES), Janssen Cilag, Lundbeck, Otsuka, Pfizer, Ferrer, Juste. The other authors declare that the research was conducted in the absence of any commercial or financial relationships that could be construed as a potential conflict of interest.
